# Direct Observation of Conversion From Walled Cells to Wall-Deficient L-Form and *Vice Versa* in *Escherichia coli* Indicates the Essentiality of the Outer Membrane for Proliferation of L-Form Cells

**DOI:** 10.3389/fmicb.2021.645965

**Published:** 2021-03-11

**Authors:** Taiki Chikada, Tomomi Kanai, Masafumi Hayashi, Taishi Kasai, Taku Oshima, Daisuke Shiomi

**Affiliations:** ^1^Department of Life Science, College of Science, Rikkyo University, Tokyo, Japan; ^2^Department of Biotechnology, Toyama Prefectural University, Toyama, Japan

**Keywords:** L-form, *Escherichia coli*, peptidoglycan, outer membrane, antibiotics

## Abstract

Gram-negative bacteria such as *Escherichia coli* are surrounded by an outer membrane, which encloses a peptidoglycan layer. Even if thinner than in many Gram-positive bacteria, the peptidoglycan in *E. coli* allows cells to withstand turgor pressure in hypotonic medium. In hypertonic medium, *E. coli* treated with a cell wall synthesis inhibitor such as penicillin G form wall-deficient cells. These so-called L-form cells grow well under anaerobic conditions (i.e., in the absence of oxidative stress), becoming deformed and dividing as L-form. Upon removal of the inhibitor, they return to the walled rod-shaped state. Recently, the outer membrane was reported to provide rigidity to Gram-negative bacteria and to strengthen wall-deficient cells. However, it remains unclear why L-form cells need the outer membrane for growth. Using a microfluidic system, we found that, upon treatment with the outer membrane-disrupting drugs polymyxin B and polymyxin B nonapeptide or with the outer membrane synthesis inhibitor CHIR-090, the cells lysed during cell deformation and division, indicating that the outer membrane was important even in hypertonic medium. L-form cells could return to rod-shaped when trapped in a narrow space, but not in a wide space, likely due to insufficient physical force. Outer membrane rigidity could be compromised by lack of outer membrane proteins; Lpp, OmpA, or Pal. Deletion of *lpp* caused cells to lyse during cell deformation and cell division. In contrast, *ompA* and *pal* mutants could be deformed and return to small oval cells even when less physical force was exerted. These results strongly suggest that wall-deficient *E. coli* cells require a rigid outer membrane to survive, but not too rigid to prevent them from changing cell shape.

## Introduction

Most bacteria are surrounded by a cell wall or peptidoglycan, a large molecule consisted glycan strands cross-linked by short peptides. Gram-negative bacteria including *E. coli* are enclosed by an inner and outer membrane, separated by a thin peptidoglycan layer. In a hypotonic medium, when peptidoglycan synthesis is inhibited by antibiotics such as penicillin, or peptidoglycan is degraded by lysozyme, bacterial cells are lysed by turgor pressure (Vollmer and Seligman, 2010; Egan et al., 2020). However, many bacteria, following inhibition of peptidoglycan synthesis, can switch to a state, the L-form, in which they proliferate without the cell wall. These cells cope well with decreased turgor pressure under hypertonic conditions and can revert to walled rod-shaped cells by restoring cell wall synthesis (Ranjit and Young, 2013; Billings et al., 2014; Kawai et al., 2014; Mercier et al., 2014). The L-form was discovered by Klieneberger in 1935 (Klieneberger, 1935, 1936). Since then, various attempts have been made to convert walled *E. coli* to the L-form under laboratory conditions, including through addition of sucrose, Mg^2+^, and penicillin (Lederberg and Clair, 1958). In recent years, conversion of the Gram-positive bacterium *Bacillus subtilis* to the L-form was shown to require enhanced membrane synthesis (Mercier et al., 2013), whereas an anaerobic environment promoted growth of the L-form (Kawai et al., 2015). Cell division of walled *E. coli* and *B. subtilis* is regulated by FtsZ, a tubulin homolog; whereas division of the *B. subtilis* L-form appears to be unregulated (Leaver et al., 2009). These findings can be extrapolated to other Gram-positive or Gram-negative bacteria, including *E. coli* (Mercier et al., 2016). Nevertheless, it remains unclear to what extent conversion of *E. coli* to the L-form depends on FtsZ and anaerobic conditions. *E. coli* L-form cells grew under aerobic conditions and required FtsZ (Joseleau-Petit et al., 2007), whereas peptidoglycan synthesis was required for their conversion and/or proliferation (Joseleau-Petit et al., 2007; Billings et al., 2014). Contrasting evidence may be explained by different experimental conditions employed, such as aeration and the antibiotics used to induce L-form conversion.

Conversion to L-form from walled cells includes several steps (Errington et al., 2016). (1) Inhibition of cell wall synthesis or degradation of cell wall in hypertonic medium induces conversion to protoplast cells, which do not proliferate. (2) Excess membrane synthesis allows the protoplast cells to induce cell shape deformation. (3) Some deformed cells can proliferate as L-form state. The mechanism required for conversion to the L-form may be more complex in Gram-negative than in Gram-positive bacteria because of the outer membrane. The outer membrane is thought to act as a barrier against substances from the external environment, while the peptidoglycan provides cell rigidity (Koch, 1988; Höltje, 1998; Deng et al., 2011; Zgurskaya et al., 2015). However, recently it was revealed that the outer membrane and outer membrane proteins, Lpp, OmpA, and Pal, which localize to the outer membrane, also contributed to cell rigidity (Rojas et al., 2018). Conversion to the L-form was lower in strains lacking these outer membrane proteins after treatment with cefsulodin (Cef), which targets the penicillin-binding proteins PBP1A and PBP1B (Rojas et al., 2018). Furthermore, mechanical rigidity of the outer membrane is required for stable L-form proliferation under low-osmotic conditions (Osawa and Erickson, 2019), as evidenced by the cell wall-deficient *E. coli* NC-7 strain (Onoda et al., 1987). Nevertheless, the following questions should be addressed. (1) Is the outer membrane required for conversion or proliferation of an unstable *E. coli* L-form via antibiotics other than Cef? (2) What is the role of the outer membrane and outer membrane proteins, such as Lpp, OmpA, and Pal, during these processes? (3) Are these factors important for reverting to walled cells?

**FIGURE 1:**
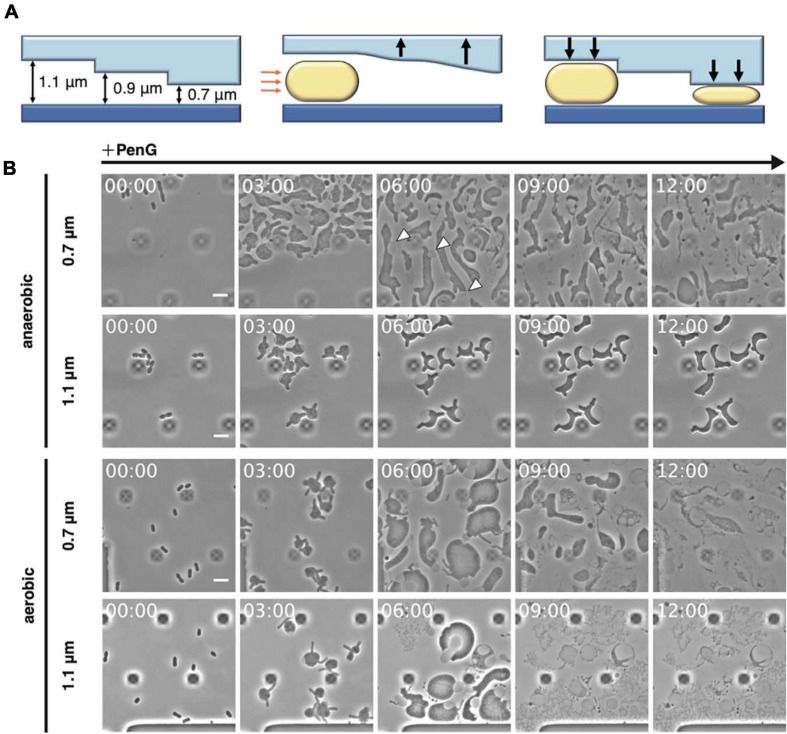
Observation of L-form formation over time using a microfluidic device. **(A)** Schematic overview of the observations conducted using the microfluidic device. Orange arrows indicate air pressure and black arrows indicate the motions of the ceiling upward and downward. Rod-shaped *E. coli* cells are shown. **(B)** Time-lapse images showing the conversion of BW25113 (WT) cells trapped at different ceiling heights under aerobic or anaerobic conditions. Cells were grown in NB/MSM medium containing PenG. Images were taken every 10 min. White arrowheads show the outer membrane. The dark square-like structures are braces for the plate of the microfluidic device. Time in each picture is shown as hour:min. Scale bar: 5 μm. Height of ceiling is 0.7 or 1.1 μm.

To clarify the effect of environmental stimuli, such as cationic ions and mechanical stress, on L-form conversion, proliferation, and return to rod-shaped cells, it is necessary to efficiently add and remove Mg^2+^ and antibiotics even under anaerobic conditions. Thus, we used a microfluidic system enabling growth of bacterial cells (Figure 1A), which was employed to observe conversion to the L-form and *vice versa* (Billings et al., 2014). Here, we could repeatedly and easily visualize conversion to the L-form in the presence of penicillin G (PenG) and fosfomycin (Fos) under anaerobic conditions obtained by replacing air with N_2_ gas. Consistent with previous reports that show the importance of the outer membrane for growth of the L-form (Rojas et al., 2018; Osawa and Erickson, 2019), we found that in a medium containing a low concentration of Mg^2+^ or outer membrane synthesis inhibitors, walled *E. coli* cells converted to the L-form, but then lysed and failed to grow. In addition, the cell deformation observed after L-form conversion was critical for these cells to proliferate and return to the rod-shaped form. The cell deformation stimulated either mechanically by the microfluidic system or genetically by the deletions of outer membrane proteins (OmpA, and Pal), which reduce the outer membrane rigidity, could stimulate proliferation of wall-deficient cells and their return to the rod-shaped type. Thus, these findings suggest that the function of the outer membrane is more complex than previously thought and that the outer membrane plays contrasting roles, offering rigidity, and preventing deformation, also enabling interconversion between L-form and rod-shaped walled cells.

## Materials and Methods

### Bacterial Strains and Growth Medium

All strains were derivatives of *E. coli* K-12 and are listed in Supplementary Table 1. BW25113 is a wild-type (WT) strain. Cells were grown in NB/MSM medium (Leaver et al., 2009) comprising 2× nutrient broth (NB) (Oxoid, United Kingdom) mixed 1:1 with 2× magnesium-sucrose-maleic acid (MSM; 40 mM MgCl_2_, 1 M sucrose, 40 mM maleic acid, pH 7.0). When cells were converted to the L-form on plates, 0.75% agar was added (NA/MSM). Antibiotics were added at the following concentrations: 300 μg/mL PenG, 400 μg/mL Fos, 100 μg/mL Cef, 50 μg/mL polymyxin B, 50 μg/mL polymyxin B nonapeptide, and 10 μM CHIR-090. 3-{[(7-hydroxy-2-oxo-2H-1-benzopyran-3-yl)carbonyl]amino}-D-alanine hydrochloride (HADA) and Alexa-Fluor 488 conjugate of wheat germ agglutinin (WGA) were added at 0.5 mM and 10 μg/mL, respectively.

### Strain Construction

Detailed methods for strain construction are described in Supplementary Materials.

### L-Form Conversion on NA/MSM Plates

Cells were grown overnight in NB/MSM at 30°C. The cell concentration was then adjusted to OD_600_ = 1.0 by NB/MSM using a Nanodrop One (Thermo Fisher Scientific, Waltham, MA, United States). Serial dilutions ranging from 10^–1^ to 10^–6^ by NB/MSM were performed and 5 μL of the 10^–2^ and 10^–6^ dilutions were inoculated on NA/MSM plates with or without antibiotics. The plates were incubated at 30°C for 3 days under anaerobic conditions using an anaeropack (MGC, Tokyo, Japan). Then, the colonies along with some agar were picked, placed on a slide, and covered by a cover slip. Samples were observed under an inverted microscope (Axio Observer; Zeiss, Jena, Germany) equipped with Objective Plan-Apochromat 100×/1.40 Ph3 (Zeiss) and filter sets 49, 38HE, and 63HE (Zeiss), and images were taken and processed using ZEN (Zeiss), Photoshop 2020 (Adobe, United States), and ImageJ (NIH, United States) software.

### L-Form/Walled Cells Conversions in a Microfluidic Device

Cells were grown to early log phase (OD_600_ = 0.35–0.45) in NB/MSM at 30°C. A 50-μL aliquot of cell culture was centrifuged, and the pellet was resuspended in 1 mL NB/MSM. Next, 50 μL of cell suspension was loaded into B04A microfluidic plates (CellASIC ONIX, Merck, United States). The air in the microfluidic plates was replaced with N_2_ by an N_2_ gas generating device (SIC, Tokyo, Japan). The microfluidic device was controlled by software supplied by the manufacturer (Merck). The microfluidic plates were placed under the microscope and observed under an inverted microscope (Zeiss) equipped with Objective Plan-Apochromat 100×/1.40 Ph3 (Zeiss) and filter sets 49, 38HE, and 63HE (Zeiss); images were taken and processed as mentioned in section 2.3. The areas of the entire cell surrounded by the outer membrane and cytoplasmic domain were measured by Image J (NIH).

## Results

### Conversion From Walled Cells to the L-Form

Inhibition of peptidoglycan synthesis stimulates conversion to the L-form in *E. coli*. As described by Lederberg and Clair (1958), conversion requires at least 0.8% agar, meat extract, Mg^2+^, sucrose, and penicillin. More recently, anaerobic conditions were shown to promote L-form proliferation (Kawai et al., 2015). To directly observe conversion from walled *E. coli* cells to the L-form and the reverse process, a microfluidic system equipped with plates for bacterial cells and allowing addition and removal of peptidoglycan synthesis inhibitor was employed (Figure 1A). NB/MSM medium, which includes NB, Mg^2+^, sucrose, and antibiotics, was used and N_2_ was pumped to ensure anaerobic conditions, unless otherwise stated. When the cell suspension was loaded on the plate, the flow pushed the plate ceiling upward. The ceiling had a stairs-like structure with the following heights: 0.7, 0.9, 1.1, 1.3, 2.3, and 4.5 μm. When the ceiling descended back toward its initial position, the cells were trapped at different heights. WT *E. coli* cells were loaded on the plate and trapped at each height, including 0.7, 0.9, and 1.1 μm, as expected. NB/MSM medium containing PenG, which inhibits the transpeptidase activity of penicillin-binding proteins, was added to induce the L-form (time 0) and administered for 24 h.

Usually, in our experience, conversion on NA/MSM plates is low (1 per 3∼5 × 10^3^ cells), consistent with previous evidence (1 per 10^3^∼10^4^ cells) (Domínguez-Cuevas et al., 2012), and growth of L-form *E. coli* in liquid medium is problematic (Mercier et al., 2014). However, most cells under anaerobic conditions in our device (approximately one every three cells) could be converted to the L-form at 0.7 μm height, whereas the rest of the cells lysed (Figure 1B anaerobic 0.7 μm, and Supplementary Movie 1). L-form cells assumed an amorphous amoeboid appearance, and divided at random positions, confirming previous observations on agarose pads (Leaver et al., 2009; Mercier et al., 2013; Mickiewicz et al., 2019; Osawa and Erickson, 2019). In our device, agarose was not required for the L-form conversion. Therefore, we suspected that trapping the cells with the ceiling might be important for their deformation and division. We could clearly observe the outer membrane surrounding L-form cells (Figure 1B, see cells indicated by arrows at 6 h), supporting the role of the outer membrane in providing rigidity to *E. coli* L-form cells (Rojas et al., 2018). Interestingly, at a height of 1.1 μm, WT cells did not convert to the ameba-like L-form, but became instead large and spherical, with a vast periplasmic space, and did not divide (Figure 1B anaerobic 1.1 μm, and Supplementary Movie 1). These results indicate that the narrow ceiling is required for conversion from walled cells to amoeboid wall-deficient cells.

Given that L-form growth is not efficient under aerobic conditions (Kawai et al., 2015), we examined which step(s) of the conversion process were promoted under anaerobic conditions (Figure 1B aerobic, and Supplementary Movie 1). Addition of PenG caused the cells to expand and deform into an amoeboid shape at 0.7 μm height under both aerobic and anaerobic conditions. However, in an aerobic environment, most cells lysed within 12 h, indicating that the cells failed to proliferate. At a height of 1.1 μm, PenG caused the cells to expand, but they lysed within 9 h (Figure 1B aerobic 1.1 μm, and Supplementary Movie 1). Quantitative analyses of cell size (μm^2^), in anaerobic and aerobic conditions at different heights, revealed the cells under aerobic conditions expanded more than under anaerobic conditions at both heights (Supplementary Table 2). However, cells under aerobic conditions were lysed. These results confirmed better L-form growth under anaerobic conditions (Kawai et al., 2015). Taken together, these findings indicated that our system could support L-form conversion.

### Reversal From L-Form to Rod-Shaped Cells

**FIGURE 2:**
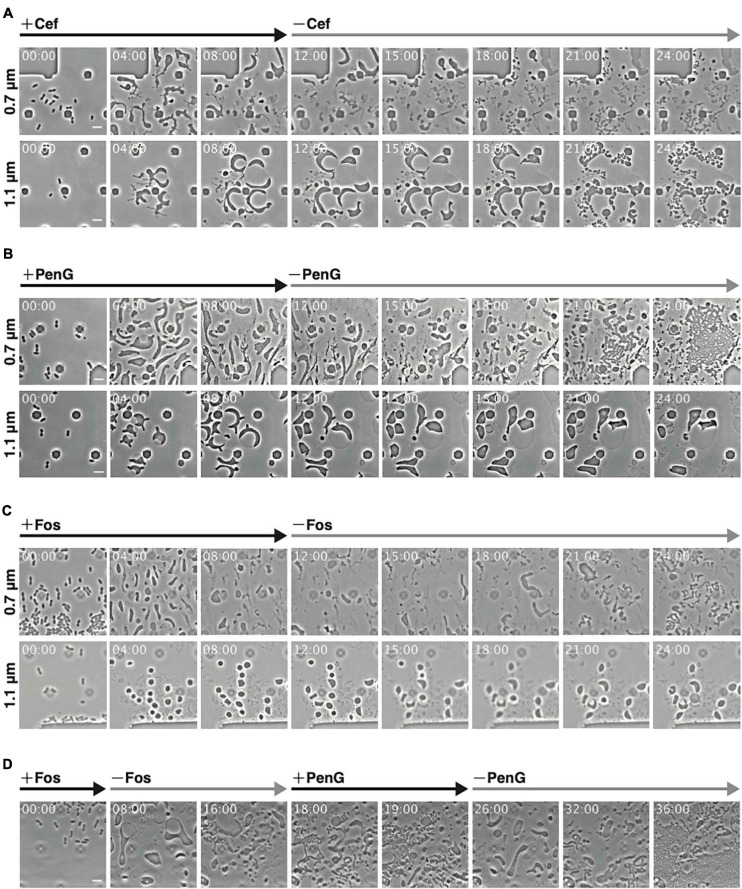
Observations of conversion and reversal between L-form and rod-shaped cells with different antibiotics at different ceiling heights. **(A–C)** Comparison of the interconversion process in the presence of different antibiotics. Cells (WT) were grown for 12 h in NB/MSM medium containing Cef **(A)**, PenG **(B)**, and Fos **(C)**, after which antibiotics were removed and the cells were grown for another 12 h. **(D)** Smooth switching of cell proliferation mode between walled cell and L-form. Cells (WT) were grown first in NB/MSM medium containing Fos for 8 h, then in medium without Fos for 10 h, followed by NB/MSM medium containing PenG for 8 h, and finally in medium without PenG for 10 h. Images were taken every 10 min. Time in each picture is shown as hour:min. Scale bars: 5 μm. Height of ceiling is 0.7 or 1.1 μm.

Using Cef allows for L-form conversion even under aerobic conditions and in liquid medium, eliminating the need for agar pads (Joseleau-Petit et al., 2007; Billings et al., 2014). As L-form *E. coli* generated by Cef could revert to rod-shaped cells under the above conditions, we examined whether the same applied to ameba-like L-form cells generated in our microfluidic system. WT cells were converted to L-form within 12 h in the presence of antibiotics (Cef, PenG, and Fos) and anaerobic conditions, followed by antibiotics removal (Figure 2). First, though, we confirmed Cef could induce conversion to the L-form at a height of 0.7 μm (Figure 2A 0.7 μm and Supplementary Movie 2), even though cell shape differed compared to PenG-induced L-form (Figure 1B). Small rod or oval cells were observed 6 h after removing Cef (time 18 h), when the first signs of cell division and a return to rod-shaped cells (time 21 h) were detected. This finding was consistent with a previous report (Billings et al., 2014). At a height of 1.1 μm, cells became large and spherical, with a vast periplasmic space (Figure 2A 1.1 μm and Supplementary Movie 2). Interestingly, after removing Cef, the cytoplasmic compartment of these cells divided into small ovoid portions while the outer membrane remained intact.

Some L-form cells generated by PenG at a height of 0.7 μm reverted to rod-shaped cells at 18 h (6 h after removal of PenG) and more such cells were observed at 21 h (Figure 2B 0.7 μm and Supplementary Movie 2). These cells appeared to recover faster than L-form cells generated by Cef. At a height of 1.1 μm, cells were large and spherical (Figure 1B), but their shape did not change, and the cytoplasm did not divide even after removal of PenG (Figure 2B 1.1 μm and Supplementary Movie 2). This suggests that surface contact or pressure might stimulate conversion to the L-form and reversal to walled cells.

We also examined conversion to the L-form and restoration of a rod shape upon treatment and removal of Fos. At a height of 0.7 μm, cells converted successfully to the L-form and then reverted to rod-shaped (Figure 2C 0.7 μm and Supplementary Movie 2) at 21 h (9 h after removal of Fos). Return from the L-form to rod-shaped cells was slower with Fos treatment than with PenG or Cef. The peptidoglycan could be synthesized using lipid II, which was generated immediately after PenG or Cef were removed. Fos inhibits MurA, which is the first enzyme in the peptidoglycan synthesis pathway, whereas PenG and Cef target later stages. This may explain why it takes longer for cells to revert to rod-shaped following Fos treatment than with PenG or Cef. At a height of 1.1 μm, Fos-induced L-form *E. coli* were deformed, large, and spherical, although smaller than those generated by PenG. Moreover, their shape did not change (Figure 2C 1.1 μm and Supplementary Movie 2).

To determine whether reverted rod-shaped cells could repeatedly convert to the L-form (Figure 2D and Supplementary Movie 3), cells at a height of 0.7 μm were first treated with Fos and converted to the L-form. They were then reverted to rod-shaped cells upon removal of Fos and converted again to ameba-like L-form cells by addition of PenG. Finally, upon removal of PenG, L-form cells reverted to rod-shaped cells. These experiments strongly indicate that cell wall-deficient cells generated by the antibiotics used in this study were alive and could return to the walled state in a narrow space.

### Peptidoglycan in L-Form Cells

It was reported that L-form cells generated by Cef retained 7% of the original peptidoglycan (Joseleau-Petit et al., 2007; Billings et al., 2014) and that ongoing peptidoglycan synthesis was required for L-form proliferation under aerobic conditions (Joseleau-Petit et al., 2007). However, Fos, which completely inhibits peptidoglycan synthesis, could also induce conversion to L-form (Figure 2; Mercier et al., 2014). Furthermore, *murC*, which is also involved in peptidoglycan synthesis, can be deleted in *B. subtilis* L-form (Kawai et al., 2014), indicating that peptidoglycan synthesis is not essential for L-form proliferation under anaerobic conditions. To examine whether L-form cells generated by PenG and Fos retained the peptidoglycan under our experimental conditions, we stained L-form cells with HADA, which replaces D-alanine in the peptidoglycan (Cava et al., 2011; Lupoli et al., 2011; Qiao et al., 2014), and WGA, which binds to N-acetylglucosamine in the peptidoglycan (Ursell et al., 2014; Kawazura et al., 2017).

**FIGURE 3:**
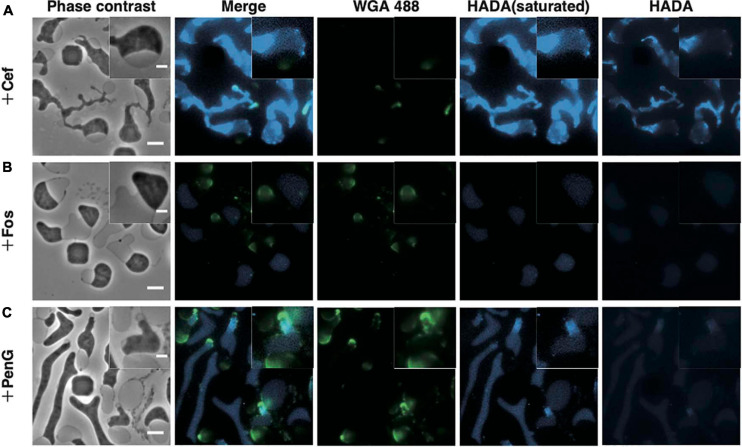
Visualization of the peptidoglycan. **(A–C)** Peptidoglycan stained with HADA and WGA. Cells were grown for 4 h in NB/MSM medium containing HADA and WGA, loaded into the microfluidic plate, and grown further in medium containing HADA, WGA, and antibiotics (Cef, Fos, and PenG). HADA and WGA were washed out for 20 min. Phase contrast, fluorescence, and merged images are shown. The fluorescence of HADA was so faint that both pictures with increased contrast and with normal contrast were shown. Magnified images are also shown. Scale bars: 5 and 2 μm (in magnified images). Height of ceiling is 0.7 μm.

Before antibiotics were added, WT cells were stained with HADA and WGA to confirm staining of the cell periphery (Supplementary Figure 1). Then, cells were treated with Cef in the presence of HADA and WGA. Because excitation light (especially 350 nm light) may be toxic and inhibit proliferation, no observation at this wavelength was made for 4 h after adding Cef. After that, cells were washed with NB/MSM medium without HADA and WGA and visualized. HADA fluorescence was detected throughout most of the entire cell periphery, but particularly in the thin-most section of L-form cells (Figure 3A). A time-lapse revealed this section was derived from the central cylinder of rod-shaped cells (Supplementary Figure 2 and Supplementary Movie 4), where the Rod complex required for peptidoglycan synthesis is localized (Kawazura et al., 2017). Because WGA fluorescence did not co-localize with HADA fluorescence (Figure 3A), we assumed the latter marked the nascent peptidoglycan, whereas the former denoted peptidoglycan synthesized before L-form conversion or peptidoglycan debris. Given that Fos blocks all peptidoglycan synthesis, if HADA stained the nascent peptidoglycan, no HADA fluorescence should be observed in L-form cells generated by Fos. This was the case, while WGA fluorescence was detected in the periplasmic space of L-form cells generated by Fos (Figure 3B). These results suggest HADA labeled the nascent peptidoglycan in the L-form and WGA labeled the peptidoglycan synthesized before conversion. It remains unclear, though, why HADA fluorescence was not observed in the periplasm with WGA and the original peptidoglycan in walled cells. Hence, as previously reported (Joseleau-Petit et al., 2007), L-form cells generated by Cef still synthesize peptidoglycan and retain some, even if incomplete, amount of it.

Next, we applied HADA and WGA staining to determine whether the L-form generated by PenG retained any peptidoglycan. WGA fluorescence was detected in the periplasm, while HADA fluorescence was detected in the portion derived from the central cylinder section of rod-shaped cells, as in the L-form generated by Cef (Figure 3C and Supplementary Movie 4). However, HADA fluorescence in the L-form generated by PenG was weaker than when generated by Cef, but stronger than that produced by Fos. This result raises two possibilities: (1) L-form cells generated by PenG do not synthesize peptidoglycan or (2) because PenG inhibits multiple penicillin-binding proteins, thus affecting replacement of HADA with D-Ala in the peptidoglycan, it is possible that the peptidoglycan was synthesized but HADA was not efficiently incorporated into it (Hsu et al., 2017; Kuru et al., 2017). In either case, we can conclude that L-form cells generated by PenG could not effectively synthesize peptidoglycan. These results indicate that, although L-form *E. coli* may be capable of synthesizing peptidoglycan, this ability is not required for survival of Gram-negative wall-deficient cells under anaerobic conditions.

### Mg^2+^ Is Required for L-Form Proliferation

**FIGURE 4:**
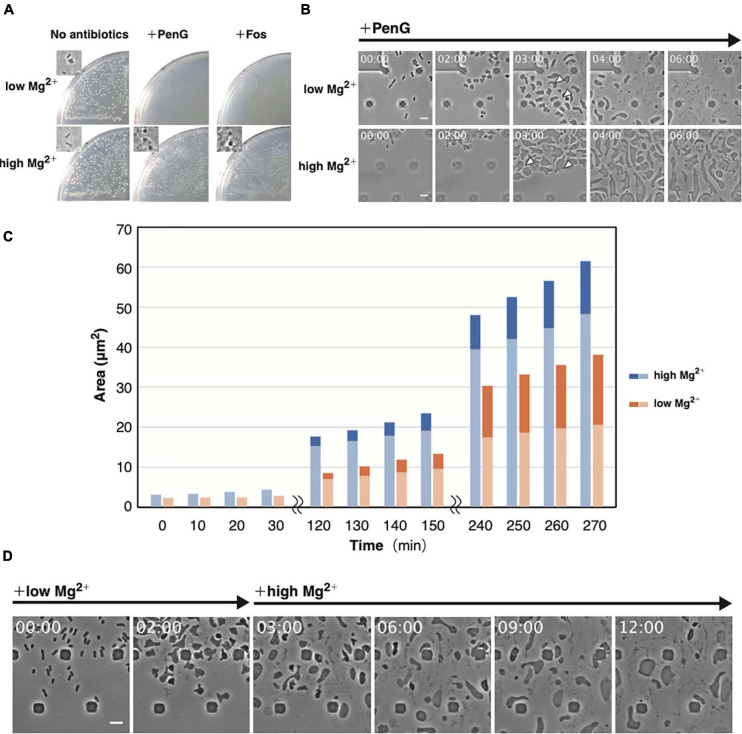
Importance of Mg^2+^ for proliferation of the L-form. **(A)** Effect of Mg^2+^ on L-form colony formation in the presence (high Mg^2+^) or absence (low Mg^2+^) of additional Mg^2+^ with or without antibiotics (PenG or Fos). Plates were incubated for 3 days at 30°C under anaerobic conditions. Phase-contrast micrograph of cells from a typical colony was shown in the inset. **(B)** Time-lapse images of conversion to the L-form in NB/MSM medium containing PenG and in the presence or absence of additional Mg^2+^. Images were taken every 10 min. Time in each picture is shown as hour:min. Arrows indicate the periplasmic space in the selected cells. **(C)** The cytoplasmic and whole-cell areas were measured from pictures taken at the indicated time points. The areas of the entire cell surrounded by the outer membrane and the cytoplasmic domain are measured by Image J. Blue and orange bars indicate cells grown in medium containing high and low Mg^2+^, respectively. The dark and light areas indicate the area of the periplasmic and the cytoplasmic regions, respectively. Areas of 10 cells were measured at each time point. **(D)** Time-lapse images of conversion to the L-form and subsequent proliferation. Cells were grown in low Mg^2+^ medium containing PenG for 3 h, after which 40 mM Mg^2+^ was added to the medium. Images were taken every 10 min. Time in each picture is shown as hour:min. Scale bars: 5 μm. Height of ceiling is 0.7 μm.

Mg^2+^ is required to generate protoplasts and L-form cells (Lederberg and Clair, 1958). Treatment with Ca^2+^ and Mg^2+^ chelators, such as EGTA or EDTA, alters the morphology of stable *E. coli* L-form NC-7 cells from an irregular angular shape to a round one (Onoda et al., 2000; Osawa and Erickson, 2019). The outer membrane is negatively charged owing to phosphate groups in the lipopolysaccharide (LPS). As divalent cations interact with these phosphate groups, they contribute to outer membrane integrity (Nikaido, 2003). EDTA was shown to reduce the number of WT *E. coli* L-form colonies on plates containing Cef under aerobic conditions (Rojas et al., 2018), highlighting the importance of the outer membrane or LPS for L-form conversion or proliferation. To determine which step(s) in these processes might be inhibited by the absence of Mg^2+^, we employed our microfluidic device. We denoted it as “low Mg^2+^”; whereas regular NB/MSM medium is referred as “high Mg^2+^” medium (Figure 4). When we prepared “low Mg^2+^” medium, we excluded Mg^2+^ from MSM. According to the manufacture’s website^1^, the NB used in this study contains “Lab-Lemco” powder (beef extract), yeast extract, peptone, and NaCl. Although the manufacturer has not disclosed how much Mg^2+^ is contained in beef extract, yeast extract, and peptone, but these compounds may contain small amounts of Mg^2+^, like LB medium (Nikaido, 2009). Based on data published by another company (BD), we estimated NB contained approximately 70 μM Mg^2+^. Thus, the “low Mg^2+^” and “high Mg^2+^” mediums contain approximately 70 μM and 20 mM Mg^2+^, respectively. First, we examined whether L-form colonies could form on a low Mg^2+^ plate in the presence of PenG or Fos. In the absence of antibiotics, colonies were observed on both low and high Mg^2+^ plates, indicating that the low Mg^2+^ medium contains enough Mg^2+^ concentration to grow, whereas in the presence of antibiotics, colonies would form only on the high Mg^2+^ plate (Figure 4A). This finding indicates that the small amount of Mg^2+^ contained in the culture medium could not yield L-form colonies. Next, we observed conversion to the L-form in low and high Mg^2+^ media. As already shown, cells changed to an ameba-like L-form and proliferated in high Mg^2+^ medium (Figure 4B and Supplementary Movie 5) but lysed after 6 h in low Mg^2+^ medium (Figure 4B and Supplementary Movie 5), suggesting that Mg^2+^ is required for proliferation of the L-form. These cells displayed a larger periplasmic space in low Mg^2+^ than in high Mg^2+^ medium (Figure 4B, 3 h).

From the images, we measured the areas of whole cells, a region surrounded by the outer membrane, and the cytoplasmic region (Figure 4C and Supplementary Figure 3). In the presence of high Mg^2+^, both the whole cell and the cytoplasm grew almost synchronously, but in the presence of low Mg^2+^, the rate of increase of the whole cell was higher than the cytoplasm. A quantitative assessment (Supplementary Figure 3) revealed that the periplasmic space made up a larger proportion of the cell area in cells grown in low Mg^2+^ medium than in those on high Mg^2+^. This result suggests that the increase in inner and outer membranes does not occur synchronously in low Mg^2+^ medium, allowing the outer membrane to become larger and weaker, making it more prone to expansion, and eventually leading to cells lysis. If Mg^2+^ is added before the cells lyse, they should continue to proliferate. To confirm this hypothesis, we added Mg^2+^ after 3 h to low Mg^2+^ medium and observed that ameba-like L-form cells were still alive at 6 h and beyond (Figure 4D and Supplementary Movie 6). This low amount of Mg^2+^ would convert cells to their amoeboid L-form, but not for their proliferation. Hence, Mg^2+^ is required to maintain outer membrane integrity, supporting proliferation of L-form cells even under anaerobic conditions.

### The Outer Membrane Is Required for L-Form Survival and Proliferation

**FIGURE 5:**
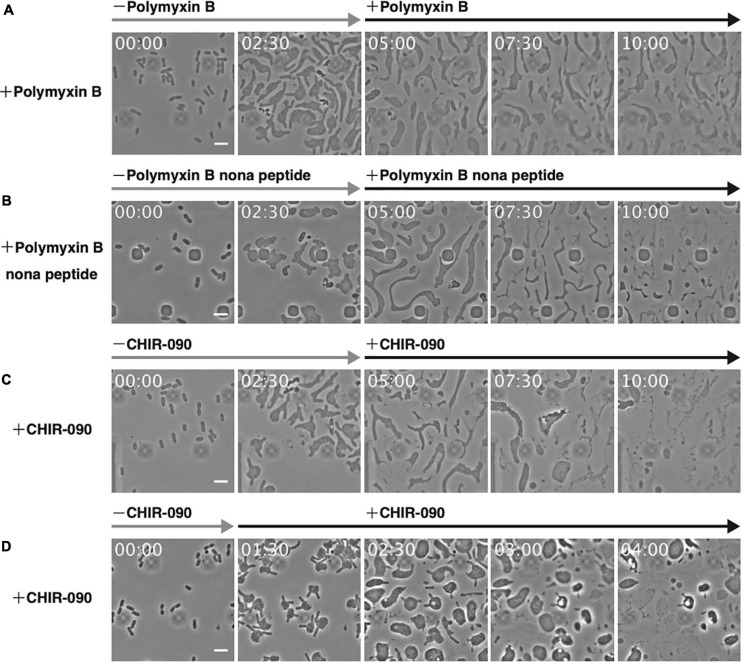
Effect of antibiotics targeting the outer membrane on L-form cells. **(A–C)** The antibiotics polymyxin B **(A)**, polymyxin B nonapeptide **(B)**, and CHIR-090 **(C)** were added 5 h after adding PenG. Images were taken every 10 min. Scale bars: 5 μm. **(D)** To assess the outer membrane for conversion to the L-form, CHIR-090 was added 1.5 h after addition of PenG. Images were taken every 10 min. Time in each picture is shown as hour:min. Scale bars: 5 μm. Height of ceiling is 0.7 μm.

The above results support the importance of Mg^2+^ for the proliferation of L-form cells under hypertonic and anaerobic conditions. Mg^2+^ provides integrity to the outer membrane or LPS but enables also the activity and assembly of various proteins, such as DNA polymerase and MreB actin (Maguire and Cowan, 2002). To confirm the role of the outer membrane in the proliferation of L-form cells, cells were grown in high Mg^2+^ medium and treated with polymyxin B. This antibiotic destroys the outer membrane by replacing Mg^2+^ bound to the phosphate group of LPS (Trimble et al., 2016). After 5 h, the inner membrane of the cell had wrinkled, and the cells appeared lysed (Figure 5A and Supplementary Movie 7), indicating that both outer and inner membranes were destroyed. To restrict the effect to the outer membrane, we used polymyxin B nonapeptide, a derivative of polymyxin B that does not affect the inner membrane (Trimble et al., 2016). Here, most cells lysed, but some remained alive (Figure 5B and Supplementary Movie 8), indicating the outer membrane maintained L-form cell integrity. Next, we investigated whether L-form proliferation was inhibited when LPS biosynthesis was blocked by treating cells with CHIR-090, an inhibitor of LpxC, a bacterial deacetylase involved in the outer membrane synthetic pathway (Barb et al., 2007). The outer membrane popped upon treatment with CHIR-090 and then L-form cells lysed (Figure 5C and Supplementary Movie 9). These results strongly suggest that the outer membrane is necessary for the proliferation of L-form cells by preventing their lysis even under hypertonic conditions. To further investigate whether the outer membrane was required for the conversion of walled cells to the L-form, we added CHIR-090 1.5 h after PenG had been administered (Figure 5D and Supplementary Movie 10). Cells swelled and converted, displaying a rabbit-like shape. Interestingly, the “ears” of the rabbit-shaped cells came off upon treatment with CHIR-090, many thread-like structures probably derived from the outer membrane were observed, and eventually the cells lysed. This result indicates that the outer membrane is required for cell survival during conversion to the L-form, and for subsequent proliferation under hypertonic and anaerobic conditions. L-form cells may have to withstand not only turgor pressure but also other unknown forces, which weaken L-form cells. Because the Gram-positive L-forms such as *B. subtilis* are covered only by cell membrane, which corresponds to the inner membrane of *E. coli* whereas the Gram-negatives are covered by inner and outer membranes, the mechanism to protect L-form cells must be different in Gram-negatives and Gram-positives.

### L-Form Conversion of *E. coli* Mutants Lacking Outer Membrane Proteins

**FIGURE 6:**
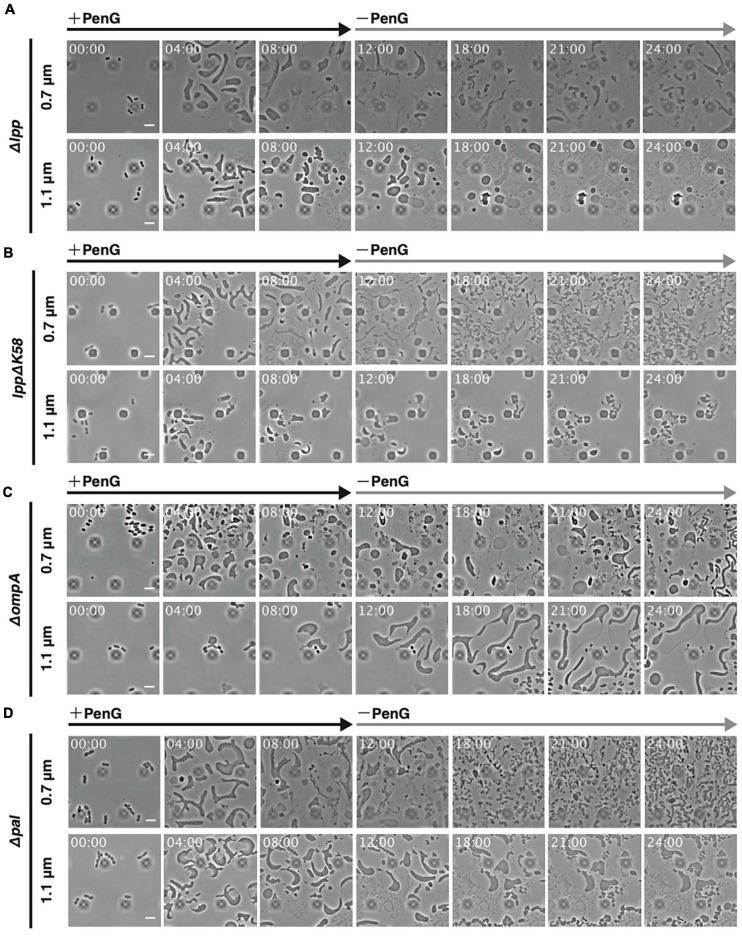
Significance of outer membrane proteins Lpp, OmpA, and Pal for conversion to the L-form and reversal to walled cells. **(A–C)** Cells lacking *lpp* **(A)**, producing *lppΔK58* **(B)**, and lacking *ompA* **(C)**, and *pal* **(D)** were trapped at a ceiling height of 0.7 or 1.1 μm. PenG was added at time 0 and was removed after 12 h. Cells were observed for 24 h and images were taken every 10 min. Time in each picture is shown as hour:min. Scale bars: 5 μm. Height of ceiling is 0.7 or 1.1 μm.

Outer membrane proteins, such as Lpp, OmpA, or Pal, interact with the peptidoglycan to maintain outer membrane integrity (Clavel et al., 1998). Of these, only Lpp is covalently linked with the peptidoglycan. Deletion of *lpp*, *ompA*, or *pal* reduced cell stiffness and mutants lacking Lpp, OmpA, or Pal displayed lower capacity to generate L-form colonies on plates containing Cef (Rojas et al., 2018). Conversion to the L-form by Cef occurs in solution and under aerobic conditions (Joseleau-Petit et al., 2007; Billings et al., 2014), suggesting that the underlying mechanism differs from that induced by PenG and Fos (see Figure 2). Here, we observed how *lpp*, *ompA*, and *pal* mutants converted to the L-form by PenG under anaerobic conditions. As shown in Figure 2B, WT cells converted to an ameba-like L-form upon treatment with PenG and then reverted to rod-shaped cells after removal of PenG at a height of 0.7 μm (Figure 2B 0.7 μm and Supplementary Movie 2), but not at a height of 1.1 μm (Figure 2B 1.1 μm and Supplementary Movie 2). Cells lacking *lpp* assumed an ameba-like L-form and some lysed. However, even those, which did not lyse, rarely reverted to a rod shape after removal of PenG at a height of 0.7 μm (Figure 6A 0.7 μm and Supplementary Movie 11). At a height of 1.1 μm, cells also acquired an amoeboid L-form but most lysed (Figure 6B 1.1 μm and Supplementary Movie 11). These results indicate Lpp plays a vital role in cell survival during conversion to the L-form and reversal to rod-shaped cells. Indeed, *Δlpp* mutants exhibit reduced outer membrane integrity (Rojas et al., 2018), which can be explained by a looser link between the outer membrane and the peptidoglycan, as Lpp binds to both. One-third of Lpp is covalently linked with the peptidoglycan (Inouye et al., 1972) while the rest non-covalently interacts with the peptidoglycan (Hancock et al., 1981; Choi et al., 1986). To confirm that the phenotype of Δ*lpp* cells was because of the lack of a link between the outer membrane and peptidoglycan by covalently attached Lpp, we observed conversion to L-form and reversion to walled state in cells producing LppΔK58. Lys 58 (K58) in Lpp is covalently linked with the peptidoglycan (Braun and Rehn, 1969) thus, LppΔK58, in which codon for Lys58 is changed to stop codon, is not linked with the peptidoglycan. This mutation induces a softening of a cell (Mathelié-Guinlet et al., 2020). At a height of 0.7 μm, *lppΔK58* cells converted to an ameba-like L-form, and some cells reverted to rod-shaped cells, although more slowly compared to WT (Figure 6B and Supplementary Movie 12). At a height of 1.1 μm, *lppΔK58* cells became spherical shape in the presence of PenG. However, upon removal of PenG, the cytoplasm of *lppΔK58* cells divided into small portions while the outer membrane remained intact (Figure 6B and Supplementary Movie 12). This phenotype differs from those of WT and Δ*lpp* cells. This result suggests that LppΔK58, a mutant Lpp which non-covalently interacts with peptidoglycan, still plays important roles in maintaining rigidity of the outer membrane and reversion to walled cells.

Cells lacking *ompA* converted to an ameba-like L-form and some could revert to rod-shaped cells at a height of 0.7 μm, although more slowly compared to WT (Figure 6C 0.7 μm and Supplementary Movie 12). Interestingly, at a height of 1.1 μm, voluminous amoeboid cells with a large periplasmic space were obtained. Upon removal of PenG, these cells became even larger, and some divided into small oval or round cells, but without reverting to fully rod-shaped cells (Figure 6C 1.1 μm and Supplementary Movie 12). This phenotype resembles *lppΔK58* cells.

As with Δ*ompA* mutants, cells lacking *pal* converted to an ameba-like L-form, and some reverted slowly to rod-shaped cells at a height of 0.7 μm (Figure 6D 0.7 μm and Supplementary Movie 13). Similarly, at a height of 1.1 μm, Δ*pal* cells assumed a large spherical shape (Figure 6D 1.1 μm and Supplementary Movie 13). Interestingly, upon removal of PenG, the cytoplasm divided into small portions while the outer membrane remained intact at 1.1 μm height. This is also like *lppΔK58* and Δ*ompA* cells. Disruption of covalent links between Lpp and the peptidoglycan, and deletion of *ompA* and *pal* genes allowed conversion to ameba-like cells and partial reversal to small cells without concomitant mechanical stress (1.1 μm height), suggesting that the outer membrane in WT cells might inhibit the return to walled cells when concomitant mechanical stress is absent, while providing rigidity to wall-deficient cells during cell deformation and proliferation.

## Discussion

Most studies have investigated the formation of L-form colonies on plates, but conversion frequency under these conditions is low and difficult to document (Domínguez-Cuevas et al., 2012). Although many experiments have been conducted using *B. subtilis*, a Gram-positive bacterium, proliferation of Gram-negative *E. coli* L-form over time has proven more difficult (Mercier et al., 2014). To our knowledge, only one report documents the L-form in a system similar to the one we used here (Billings et al., 2014). Here, we report successfully visualizing the conversion of *E. coli* to the L-form and their return to rod-shaped cells under anaerobic conditions. With our system, conversion from walled cells to the L-form and *vice vers*a could be observed repeatedly in real time, including following the application of different antibiotics and media. Furthermore, conversion efficiency was substantially higher than what reported on plates (approximately 10^–3^ to 10^–4^ cells). Therefore, the proposed system offers the best means for visualizing conversion to the L-form.

**FIGURE 7:**
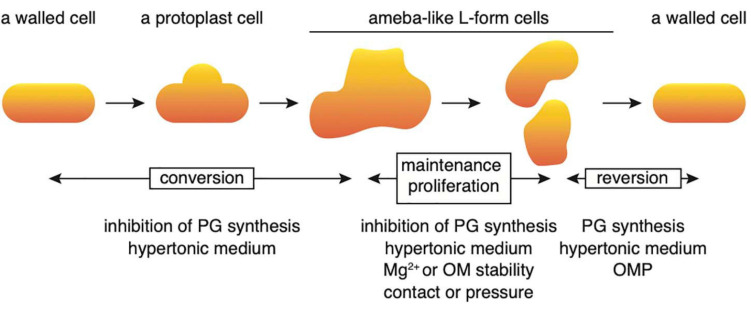
Schematic illustration of the processes regulating conversion to the L-form and reversal to a rod-shaped cell. Details are described in the text. OM, outer membrane; OMP, outer membrane protein; PG, peptidoglycan.

Conversion to the L-form and recovery to walled cells could be divided into several stages (Figure 7). First, when cell wall synthesis is inhibited by antibiotics such as PenG, Fos or Cef in a hypertonic medium, the cells swell from the center of the cell and deform, assuming an ameba-like L-form. These cells can proliferate. When cell wall synthesis is no longer inhibited, the original walled cells are restored. In this study, the factors required for each stage were identified. First, we show that no extra Mg^2+^ is necessary to transform walled cells into their amoeboid counterparts; the small amount (approximately 70 μM) of Mg^2+^ contained in NB/MSM medium is sufficient. However, Mg^2+^ must be added to the medium to enable successful proliferation of L-form cells. A rigid outer membrane is required for the maintenance and proliferation of ameba-like L-form cells and the rigidity of the outer membrane is reduced in cells grown in low Mg^2+^ medium (Nikaido, 2003) or in mutants devoid of outer membrane proteins (Rojas et al., 2018).

One feature of our system is the ability to trap cells at different ceiling heights. WT cells trapped at a height of 0.7 μm converted to ameba-like L-form cells in the presence of PenG and returned to rod-shaped cells upon PenG removal. In contrast, WT cells trapped at a height of 1.1 μm were transformed into a wall-deficient form but did not acquire an amoeboid appearance and failed to return to a rod-shaped form following removal of PenG. This finding suggests that contact between cells and solids or mechanical stress is essential for the interconversion between walled cells and the L-form, as well as for proliferation of the latter. The role of mechanical stress is also supported by the observation that elongated L-form cells grown in a narrow channel assumed a spherical shape when they were placed in a wide channel (Wu et al., 2020). This result may explain why L-form cells, absent from suspensions containing PenG and L-form colonies, appear inside the plate rather than on its surface (Joseleau-Petit et al., 2007). Some suggested that Fos inhibits the cell wall synthesis and activate fatty acid synthesis, which increase the cell membrane synthesis. Therefore, the increasing cell surface/volume ratio induces the L-form cell deformation and division (Mercier et al., 2013). However, WT cells trapped at a height of 1.1 μm were neither transformed into an amoeboid shape nor made a large periplasmic space, while keeping a small spherical shape in the presence of Fos, unlike in the presence of Cef or PenG. These indicate that the synthesis of outer and inner membranes, in Fos treated wall-deficient cells, was not enough to increase the cell surface/volume ratio to induce the L-form cell deformation in the space with a height of 1.1 μm. It was indicated that Fos reduces the cell surface/volume ratio of the walled bacterial cells (Harris and Theriot, 2016; Si et al., 2017). When the cell wall was removed from Fos treated cells, the cells would form small spherical cells by reducing its cell surface/volume ratio (Mercier et al., 2013). We suspect that the small spherical Fos treated wall-less cells might have less mechanical stress at a height of 1.1 μm and thus did not transform into an amoeboid shape and have large a periplasmic space.

Lpp, OmpA, and Pal outer membrane proteins provide stiffness (Rojas et al., 2018; Mathelié-Guinlet et al., 2020) and connect the outer membrane to the cell wall (Mathelié-Guinlet et al., 2020), explaining why *lpp*, *ompA*, and *pal* mutants failed to return to rod-shaped cells at the same speed as WT cells at a height of 0.7 μm. PBP1B and Lpp are required for recovery from lysozyme-induced spheroplasts to rod-shaped cells (Ranjit and Young, 2013; Ranjit et al., 2017). PBP1B forms a complex with TolA (Gray et al., 2015), which then joins with Pal via TolB to generate the Tol-Pal complex. The Tol-Pal complex is required for constriction of the outer membrane during cell division (Gerding et al., 2007; Szczepaniak et al., 2020). Pal interacts with OmpA (Palva, 1979). Therefore, the outer membrane lipoproteins complexed with cell wall synthesis-enzymes are important for restoring walled rod-shaped cells, and for constriction of the outer membrane during division in walled cells. Importantly, cells lacking *ompA* or *pal* could convert to L-form cells under our conditions, suggesting that the function of the outer membrane is more complex than previously thought and that the outer membrane does not only offer rigidity and prevent deformation, but also enables interconversion between L-form and rod-shaped walled cells.

Interestingly, in cells producing *lppΔK58* or lacking *ompA* or *pal*, the cytoplasm divided into small portions while the outer membrane remained intact at 1.1 μm height. Notably, an “outer membrane” surrounding these small portions could not be excluded. LppΔK58, OmpA, and Pal non-covalently interact with peptidoglycan so that cells producing LppΔK58 or cells lacking *ompA* or *pal* should reduce link between peptidoglycan and outer membrane. To explain this interesting phenotype, we hypothesized that under normal circumstances the inner membrane (plus peptidoglycan) and outer membrane divided in a coordinated fashion mediated by link between peptidoglycan and outer membrane by Lpp, OmpA, and Pal and the Tol-Pal system (Gerding et al., 2007; Yakhnina and Bernhardt, 2020); however, in cells producing *lppΔK58* and lacking *ompA*, or *pal*, the outer membrane is not correctly linked with peptidoglycan during the reversion. This result suggest that inner membrane (plus peptidoglycan) is capable of division independent of outer membrane and any external mechanical forces. If outer membrane is properly linked with peptidoglycan in WT cells without any external mechanical forces, cells are not able to divide probably because inner membrane is pulled by outer membrane. Therefore, WT cells remained spherical. We speculate that at low ceiling height, both membranes may divide simultaneously probably because of the outer membrane being physically pushed toward the inner membrane and their common subsequent fate. Once the cells return to a rod shape, they can divide normally again. As such phenotype resembles restoration of rod-shaped cells from L-form following Cef treatment at a height of 1.1 μm (Figure 2A), we hypothesize Cef may affect the link between the inner and outer membranes, as well as the transpeptidase activity of PBP1A and PBP1B, possibly thorough interaction with the Tol-Pal complex (see the above discussion). At a height of 1.1 μm, WT wall-deficient cells assumed a spherical rather than ameba-like form, which prevented their further deformation. In contrast, Δ*ompA* and Δ*pal* cells acquired an amoeboid form and became deformed. Such different output suggests that continuous cell deformation is critical for wall-deficient cells to proliferate and divide with or without antibiotics. Understanding the exact relationship between deformation and cell division will require further analysis.

In this study, we show that the outer membrane is important for growth of the *E. coli* L-form. The stable NC-7 *E. coli* L-form has been shown to withstand low-osmotic pressure due to its rigid outer membrane (Osawa and Erickson, 2019) and a lower rigidity decreased conversion to the L-form on plates (Rojas et al., 2018). However, these studies did not distinguish between conversion and subsequent L-form proliferation. Direct observation via our microfluidic system pointed to the outer membrane being critical for proliferation, as demonstrated by the ability to form ameba-like cells even in a low Mg^2+^ environment or in the absence of outer membrane proteins such as *lpp*. If the conversion from walled cells to the L-form requires membrane flexibility, a slight decrease in rigidity of the outer membrane is not a problem. However, to maintain the L-form and divide (i.e., to constrict the membrane independently of the cytoskeletal protein FtsZ), sufficient outer membrane rigidity must be provided.

In Gram-positive *B. subtilis*, membrane composition and fluidity are important for proliferation of the L-form (Mercier et al., 2012). Excessive membrane synthesis promotes deformation and division of L-form cells (Mercier et al., 2013). Even in *E. coli*, L-form cells are larger than walled cells, suggesting that membrane synthesis is enhanced. However, because *E. coli* is surrounded by both an outer and an inner membrane, it is necessary not only to promote the synthesis of the membranes but also to do so in a coordinated manner. For example, when comparing cells under low Mg^2+^ and high Mg^2+^conditions, the latter appeared larger (Figure 4B), but the proportion of periplasmic space was smaller than in cells exposed to low Mg^2+^ (Figure 4C). This is probably because under low Mg^2+^, synthesis of the inner and outer membranes is uncoordinated; whereas under high Mg^2+^, overall membrane synthesis is increased yet balanced to some extent.

Do L-form cells have a cell wall? If so, is the cell wall necessary for proliferation of the L-form? As shown previously, *murC* and *uppS*, which are essential for cell wall synthesis, could be deleted in L-form *B. subtilis* cells (Kawai et al., 2014). We have constructed a strain, in which expression of *murA*, the first protein in the cell wall synthesis pathway, is induced by arabinose (Chikada et al., manuscript in preparation). This strain can grow as walled cells only in the presence of arabinose in a normal medium, and as L-form in the absence of arabinose in the hypertonic medium. Cell wall synthesis is probably not occurring in this mutant. In the present study, HADA fluorescence was hardly observed in L-form cells generated by Fos (which inhibits MurA activity). These results indicate that L-form cells do not depend on cell wall synthesis, despite whether they are Gram-positive or Gram-negative. *E. coli* L-form cells were reported to contain peptidoglycan, with 7% of walled cells, and *murA* was required for L-form cells generated by both Cef and PenG (Joseleau-Petit et al., 2007). In addition, peptidoglycan precursors are synthesized and glycan chains are linked, but incomplete peptidoglycans are formed in the stable L-form of *Listeria monocytogenes* (Studer et al., 2016). It remains to be determined whether *murA* is required for growth of *E. coli* L-form cells. Our HADA experiments revealed that the L-form generated by Cef or PenG synthesized (incomplete) peptidoglycan, whereas L-form cells generated by Fos exhibited almost no synthesis. Therefore, we conclude that, although the L-form may synthesize peptidoglycan, peptidoglycan in L-form is incomplete and peptidoglycan synthesis is not essential for L-form conversion and proliferation under our experimental conditions.

Another open question is whether monoderm or diderm cells appeared first during evolution (Megrian et al., 2020). The recent phylogenic analysis of diderm firmicutes *Negativicutes* and *Halanaerobiales* supports the diderm first hypothesis (Antunes et al., 2016). Our results show that if a cell does not possess an outer membrane, its integrity cannot be maintained and lyses even in hypertonic medium. In contrast, if a cell does not have a cell wall, it can become deformed and rely on the outer membrane for survival and proliferation. Wall-deficient diderm cells could resist turgor pressure in low-osmotic medium (Osawa and Erickson, 2019), suggesting that even if ancient diderm cells did not possess a cell wall, they had sufficient integrity to withstand various environmental stresses including low osmolarity. At the same time, *B. subtilis* L-form cells can proliferate without an outer membrane, supporting that ancient cells might have proliferated without cell wall and outer membrane (Errington et al., 2016). L-form *B. subtilis* cells have a single phospholipid membrane, but it is not known whether they lack all cell surface components such as lipoteichoic acids. We showed that cytoplasmic region (and possibly peptidoglycan) was able to divide while outer membrane remains intact in cells producing *lppΔK58* and cells lacking *ompA* and *pal*. This result may suggest to us how diderm cells evolved into monoderm cells, that is, if diderm cells lose a link between peptidoglycan and outer membrane, the cytoplasm surrounded by inner membrane and peptidoglycan can divide independently of the outer membrane in diderm cells, and after that if they lose the outer membrane, the cells consist of the cytoplasm surrounded by inner membrane and peptidoglycan such as Gram-positives. Another conundrum regarding the early stage of evolution is whether it was the diderm cells or the cell wall that appeared first. Interestingly, the L-form generated by Fos seems smaller than those generated by PenG or Cef. These latter two could synthesize the cell wall, even if it were incomplete. In the process of evolution, cells had acquired an ability to synthesize cell walls, although they were incomplete, to withstand various environmental conditions such as high osmolarity; then grew as large spherical cells. Another hypothesis is that the surface area of cells increased to hold larger genomic DNA and intake more nutrients into the cells. To grow and maintain a large spherical shape, it may have acquired and continuously synthesized a cell wall. In either case, L-form may be thought of as mimicking the proliferation and morphology of cells in the initial stages of evolution. Then, having acquired the cell wall, it might have become possible for cells to maintain a certain shape and divide in a more controlled way.

Finally, it should be mentioned that we have constructed an experimental system for visualizing conversion to the L-form, its proliferation, and its recovery to walled cells and repeatedly. To date, the screening of mutants that do not convert to the L-form (Glover et al., 2009) may not determine which process was abnormal. Our system can clarify the detailed molecular mechanism of L-form conversion, proliferation, and recovery to walled cells, as well as answer unresolved evolutionary questions.

## Data Availability Statement

The original contributions presented in the study are included in the article/Supplementary Material, further inquiries can be directed to the corresponding author/s.

## Author Contributions

TC, TK, MH, TO, and DS made contributions to the design of the study, the acquisition, analysis, and interpretations of the data. TK analyzed the data. TC, TO, and DS made contributions to writing of the manuscript. All authors contributed to the article and approved the submitted version.

## Conflict of Interest

The authors declare that the research was conducted in the absence of any commercial or financial relationships that could be construed as a potential conflict of interest.

## Footnotes

1 http://www.oxoid.com/UK/blue/prod_detail/prod_detail.asp?pr=CM0001&c=UK&lang=EN
