# Supplementary material for: Direct Observation of Conversion From Walled Cells to Wall-Deficient L-Form and Vice Versa in Escherichia coli Indicates the Essentiality of the Outer Membrane for Proliferation of L-Form Cells
Source: Front Microbiol. 2021 Mar 11;12:645965. doi: 10.3389/fmicb.2021.645965 (PMC7991099; doi:10.3389/fmicb.2021.645965)
Supplement: Supplementary file 14 [file Data_Sheet_1.PDF]

Supplementary Material

Direct observation of conversion from walled cells to wall-deficient L-form and *vice versa* in *Escherichia coli* indicates the essentiality of the outer membrane for proliferation of L-form cells

Taiki Chikada[1], Tomomi Kanai[1], Masafumi Hayashi[1], Taishi Kasai[1], Taku Oshima[2*], Daisuke Shiomi[1*]

[1] Department of Life Science, College of Science, Rikkyo University, 3-34-1 Nishi Ikebukuro, Toshima-ku, Tokyo 171-8501, Japan

[2] Department of Biotechnology, Toyama Prefectural University, 5180, Kurokawa, Imizu, Toyama 939-0398, Japan.

1 Supplementary Methods

1.1 Strain construction

RU1637 (*Δpal::kan*) A P1 lysate prepared form JW0731 (*Δpal::kan*) was used to transduce *Δpal::kan* into BW25113 to yield RU1637.

RU1638 (*ΔompA::kan*) A P1 lysate prepared form JW0940 (*ΔompA::kan*) was used to transduce *ΔompA::kan* into BW25113 to yield RU1638.

RU1639 (*Δlpp::kan*) A P1 lysate prepared form JW1667 (*Δlpp::kan*) was used to transduce *Δlpp::kan* into BW25113 to yield RU1639.

RU2177 (*lppΔK58*) To replace the codon for Lys 58 with stop codon and insert CAT gene, which is flanked by FRT sites, into just after the stop codon, pKD3 was amplified using primers 2203 and 2204. The PCR product was introduced into strain BW25113 (WT) carrying pKD46 by electroporation, yielding RU2177 (*lppΔK58*). The sequences of primers 2203 and 2204 were ACGCAGCTCGTGCTAACCAGCGTCTGGACAACATGGCTACTAAATACCGCTAATAAGTG TAGGCTGGAGCTGCTTC (2203) and CAGACAAAAAAAATGGCGCACAATGTGCGCCATTTTTCACTTCACAGGTACTACATATG AATATCCTCCTTAG (2204), respectively.

2 Supplementary Figures and Tables

2.1 Supplementary Figures

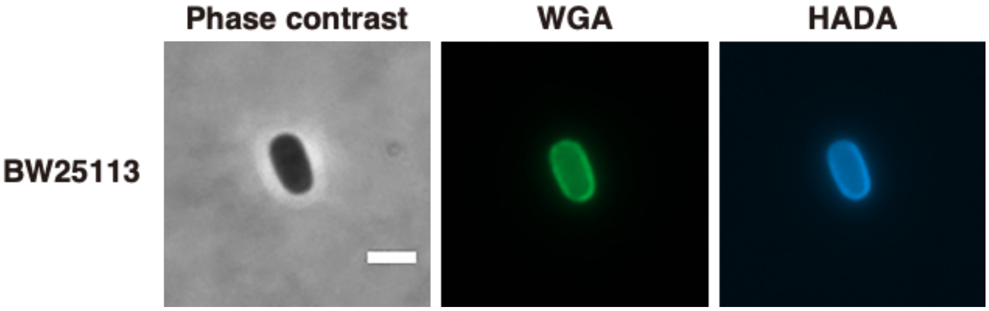

Supplementary Figure 1. Peptidoglycan staining by WGA and HADA. Phase contrast and fluorescent images are shown.

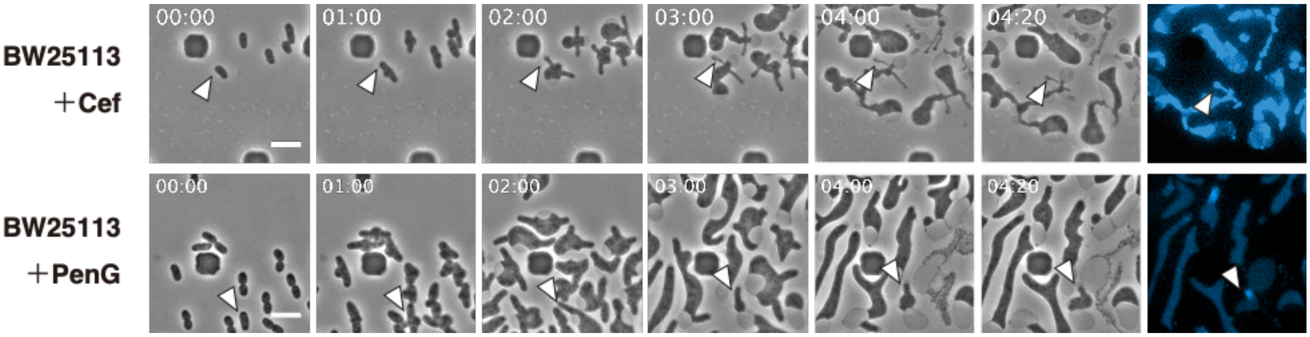

Supplementary Figure 2. Time-lapse observation of conversion to L-form by Cef and PenG. Phase contrast and fluorescent images are shown. Arrowheads indicate the cylindrical portion of the original rod-shaped cell. Scale bar is 5 μm.

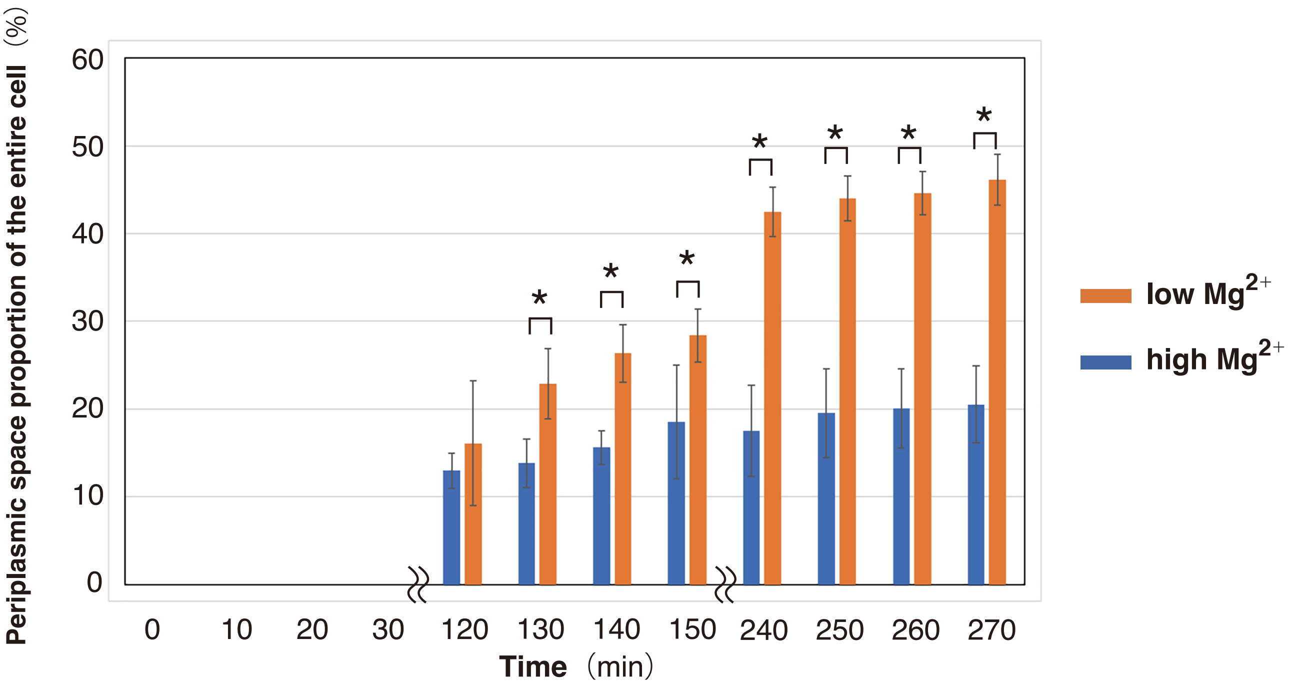

Supplementary Figure 3. The periplasmic space portion of L-form cells in low and high Mg^{2+} medium. The periplasmic and whole-cell areas were measured from pictures taken at the indicated time points. The ratio between the periplasmic and the whole-cell area is shown. Blue and orange bars indicate cells grown in medium containing high and low Mg^{2+}, respectively. Average and standard deviations are shown (n = 10). P-values were determined by unpaired T-test. $P < 0.05$ was considered significantly different, which were shown by asterisks, at the indicated time point.

2.2 Legends to Supplementary Movies

Supplementary Movie 1 (Related to Figure 1B) Time-lapse images showing the conversion of BW25113 (WT) cells trapped at different ceiling heights under aerobic or anaerobic conditions. (Top left) anaerobic at height of 0.7 μm. (Top right) anaerobic at height of 1.1 μm. (Bottom left) aerobic at height of 0.7 μm. (Bottom right) aerobic at height of 1.1 μm.

Supplementary Movie 2 (Related to Figure 2A, 2B, and 2C) Comparison of the interconversion process in the presence of different antibiotics. (Top left) +Cef at height of 0.7 μm. (Top right) +Cef at height of 1.1 μm. (Middle left) +PenG at height of 0.7 μm. (Middle right) +PenG at height of 1.1 μm. (Bottom left) +Fos at height of 0.7 μm. (Bottom right) + Fos at height of 1.1 μm.

Supplementary Movie 3 (Related to Figure 2D) Smooth switching of cell proliferation mode between walled cell and L-form.

Supplementary Movie 4 (Related to Figure 3A and 3C) Time-lapse images showing the conversion of BW25113 (WT) cells in the presence of Cef (left) or PenG (right).

Supplementary Movie 5 (Related to Figure 4B) Time-lapse images of conversion to the L-form in NB/MSM medium containing PenG and in the presence or absence (right) of additional Mg^{2+} (left).

Supplementary Movie 6 (Related to Figure 4D) Time-lapse images of conversion to the L-form and subsequent proliferation.

Supplementary Movie 7 (Related to Figure 5A) Effect of polymyxin B on L-form cells.

Supplementary Movie 8 (Related to Figure 5B) Effect of polymyxin B nonapeptide on L-form cells.

Supplementary Movie 9 (Related to Figure 5C) Effect of CHIR-090 on L-form cells.

Supplementary Movie 10 (Related to Figure 5D) Effect of CHIR-090 on conversion process to L-form cells.

Supplementary Movie 11 (Related to Figure 6A and 6B) (top) Time-lapse images showing the conversion of Δ*lpp* cells trapped at height of 0.7 μm (left) and 1.1 μm (right). (bottom) Time-lapse images showing the conversion of *lppΔK58* cells trapped at height of 0.7 μm (left) and 1.1 μm (right).

Supplementary Movie 12 (Related to Figure 6C) Time-lapse images showing the conversion of Δ*ompA* cells trapped at height of 0.7 μm (left) and 1.1 μm (right).

Supplementary Movie 13 (Related to Figure 6D) Time-lapse images showing the conversion of Δ*pal* cells trapped at height of 0.7 μm (left) and 1.1 μm (right).

2.3 Supplementary Table

Supplementary Table S1. Strains and a plasmid used in this study.

Strain	Relevant genotypes	References
BW25113	WT	(Baba et al., 2006)
JW0731 (KEIO collection)	Δ*pal::kan*	(Baba et al., 2006)
JW0940 (KEIO collection)	Δ*ompA::kan*	(Baba et al., 2006)
JW1667 (KEIO collection)	Δ*lpp::kan*	(Baba et al., 2006)
RU1637	BW25113 Δ*pal::kan*	This study
RU1638	BW25113 Δ*ompA::kan*	This study
RU1639	BW25113 Δ*lpp::kan*	This study
RU2177	BW25113 *lppΔK58 cat*	This study
**Plasmid**	**Relevant genotypes**	**References**
pKD3	*FRT-cat-FRT*, Cm^R Amp^R	(Datsenko and Wanner, 2000)
pKD46	Lambda Red recombinase, Amp^R	(Datsenko and Wanner, 2000)

Supplementary Table S2. Cells size* of *E. coli* treated with PenG in the microfluidic system (Related to Figure 1).

	Height (μm)	Time (h)	
		0	3
anaerobic	0.7	3.31 (n=10)	31.82 (n=10)
	1.1	3.02 (n=10)	17.69 (n=10)
aerobic	0.7	2.66 (n=10)	17.40 (n=10)
	1.1	3.06 (n=10)	22.42 (n=10)

*Cell size was expressed as cell area (μm^2).

3 Supplementary References

Baba, T., Ara, T., Hasegawa, M., Takai, Y., Okumura, Y., Baba, M., et al. (2006). Construction of *Escherichia coli* K-12 in-frame, single-gene knockout mutants: the Keio collection. *Mol. Syst. Biol.* 2, 2006.0008. doi:10.1038/msb4100050.

Datsenko, K. A., and Wanner, B. L. (2000). One-step inactivation of chromosomal genes in *Escherichia coli* K-12 using PCR products. *Proc. Natl. Acad. Sci. U.S.A.* 97, 6640–6645. doi:10.1073/pnas.120163297.
